# Editorial: Functional Brain Mapping of Epilepsy Networks: Methods and Applications

**DOI:** 10.3389/fnins.2019.00417

**Published:** 2019-05-21

**Authors:** David F. Abbott, John S. Archer, Patrick W. Carney, David N. Vaughan, Graeme D. Jackson

**Affiliations:** ^1^The Florey Institute of Neuroscience and Mental Health, Austin Hospital, Melbourne, VIC, Australia; ^2^Faculty of Medicine, Dentistry and Health Sciences, The University of Melbourne, Melbourne, VIC, Australia; ^3^Eastern Health Clinical School, Monash University, Melbourne, VIC, Australia

**Keywords:** epilepsy, fMRI, functional connectivity, default mode network, interictal epileptiform discharge, EEG, computational modeling and simulation, seizure prediction

## Introduction

This multidisciplinary Research Topic is a collection of contemporary advances in neuroimaging applied to mapping functional brain networks in epilepsy. With technology such as simultaneous electroencephalography and functional magnetic resonance imaging (EEG-fMRI) now more readily available, it is possible to non-invasively map epileptiform activity throughout the entire brain at millimeter resolution. This Research Topic includes original research studies, technical notes and reviews of the field. Due to the multidisciplinary nature of the domain, the Research Topic spans two journals: Frontiers in Neurology (Section: *Epilepsy*) and Frontiers in Neuroscience (Section: *Brain Imaging Methods)*.

In this editorial we consider the outcomes of the multidisciplinary work presented in the Research Topic. With the benefit of time elapsed since the original papers were published, we can see that the works are making a substantial impact in the field. At the time of writing, this Research Topic had well over 28,000 full-paper downloads (including over 18,500 for the 15 papers in the Epilepsy section, and over 9,500 for the 8 papers in the Brain Imaging Methods section). Several papers in the Research Topic have climbed the tier in Frontiers and received an associated invited commentary, demonstrating there is substantial interest in this research area.

## Reviews

The Research Topic's review papers set the scene for the original research papers and synthesize contemporary thinking in epilepsy research and neuroimaging methods. We see that Epilepsy, whether of a “generalized” or “focal” origin, is increasingly recognized as a disorder of large-scale brain networks. At one level it is self-evident that otherwise healthy functional networks are recruited during epileptic activity, as this is what generates patient perceptions of their epileptic aura. For example, the epileptic aura of mesial temporal lobe epilepsy (MTLE) can include an intense sensation of familiarity (déjà vu) associated with involvement of the hippocampus, and unpleasant olfactory auras which may reflect involvement of adjacent olfactory cortex. As seizures spread more widely throughout the brain, presumably along pre-existing neural pathways, patients lose control of certain functions; for example, their motor system in the case of generalized convulsions, or aspects of awareness in seizures that remain localized to non-motor brain regions. Yet these functions return when the seizure abates, implying involved brain regions are also responsible for normal brain function. What has been less clear, and difficult to investigate until the advent of functional neuroimaging, is precisely which brain networks are involved (especially in “generalized” epilepsy syndromes), and the extent to which functional networks are perturbed during seizures, inter-ictal activity, and at other times.

Functional imaging evidence of brain abnormalities in temporal lobe epilepsy is explored in Caciagli et al., including evidence of dysfunction in limbic and other specific brain networks, as well as global changes in network topography derived from resting-state fMRI. Archer et al. systematically review the functional neuroimaging of a particularly severe epilepsy phenotype, Lennox-Gastaut Syndrome (LGS), illustrating well how different forms of brain pathology can manifest in a similar clinical phenotype, simply by the nature of the healthy networks that the underlying pathology perturbs. Similarly, the mechanisms of absence seizure generation are reviewed by Carney and Jackson, revealing that it too has a signature pattern of large-scale functional brain network perturbation. The ability to make such observations has considerable clinical significance, as highlighted in the review by Pittau et al..

The tantalizing proposition that there may be a common treatment target for all focal epilepsy phenotypes is also explored in a review of the piriform cortex by Vaughan and Jackson. The piriform cortex was first implicated as a common brain region associated with spread of interictal discharges in focal epilepsy in an experiment that analyzed the spatially normalized functional imaging data of a heterogeneous group of focal epilepsy patients (Laufs et al., 2011). This finding, since replicated (Flanagan et al., 2014), led Vaughan and Jackson to explore in detail what is known of the piriform cortex. Their findings reveal the piriform has several features that likely predispose it to involvement in focal epilepsy, and features that also explain many of the peculiar symptoms experienced by patients, from olfactory auras to the characteristic nose-wiping that many patients perform postictally. This work points to the need for future studies to determine whether the piriform might be an effective target for deep brain stimulation or other targeted therapy to prevent the spread of epileptiform activity.

## Original Research

Temporal lobe epilepsy is investigated in several papers in this topic. One of these studies also introduces a new exploratory method, *Shared and specific independent component analysis* (SSICA), that builds upon independent component analysis to perform between-group network comparison (Maneshi et al.). In application to MTLE and healthy controls, three distinct reliable networks were revealed: two that exhibited increased activity in patients (a network including hippocampus and amygdala bilaterally, and a network including postcentral gyri and temporal poles), and a network identified as specific to healthy controls (i.e., effectively decreased in patients, consisting of bilateral precuneus, anterior cingulate, thalamus, and parahippocampal gyrus). These finding give mechanistic clues to the cognitive impairments often reported in patients with MTLE. Further clues are revealed in a study of the dynamics of fMRI and its functional connectivity (Laufs et al.). Compared to healthy controls, temporal variance of fMRI was seen to be most increased in the hippocampi of TLE patients, and variance of functional connectivity to this region was increased mainly in the precuneus, the supplementary and sensorimotor, and the frontal cortices. More severe disruption of connectivity in these networks during seizures may explain patients' cognitive dysfunction (Laufs et al.). Yang and colleagues also show that it may be possible to use fMRI functional connectivity to lateralise TLE (Yang et al.), which could be a useful clinical tool.

Mechanistic explanations of symptomatology beyond the seizure onset zone can also be revealed with conventional nuclear medicine techniques such as ^18^F-FDG-PET. This is demonstrated in a study of Occipital Lobe Epilepsy by Wong and colleagues, who observed that patients with automatisms have metabolic changes extending from the epileptogenic occipital lobe into the ipsilateral temporal lobe, whereas in patients without automatisms the ^18^F-FDG-PET was abnormal only in the occipital lobe (Wong et al.).

The clinical significance of the ability to non-invasively study functional brain networks extends to understanding the impact of surgery on brain networks. This Frontiers Research Topic includes an investigation by Doucet and colleagues revealing that temporal lobe epilepsy and surgery selectively alter the dorsal, rather than the ventral, default-mode network (Doucet et al.).

Another approach to better understand the mechanisms of seizure onset and broader symptomatology is computational modeling. Such an approach can track aspects of neurophysiology that cannot be readily measured: for example effective connectivity and mean membrane potential dynamics are shown by Freestone et al. to be estimable using model inversion. In a proof-of-principle experiment with simulated data, they demonstrate that by tailoring the model to subject-specific data, it may be possible for the framework to identify a seizure onset site and the mechanism for seizure initiation and termination. Also in this Research Topic, Petkov and colleagues utilize a computational model of the transition into seizure dynamics to explore how conditions favorable for seizures relate to changes in functional networks. They find that networks with higher mean node degree are more prone to generating seizure dynamics in the model, thus providing a mathematical mechanistic explanation for increasing node degree causing increased ictogenicity (Petkov et al.).

Seizure prediction is an area of considerable research, and in this Research Topic Cook and colleagues reveal intriguing characteristics in the long-term temporal pattern of seizure onset. They confirmed that human inter-seizure intervals follow a power law, and they found evidence of long-range dependence. Specifically, the dynamics that led to the generation of a seizure in most patients appeared to be affected by events that took place much earlier (as little as 30 min prior and up to 40 days prior in some patients) (Cook et al.). The authors rightly note that this information could be valuable for individually-tuned seizure prediction algorithms.

Several methodological papers in this Frontiers Research Topic prove there remains considerable potential to improve neuroimaging methods as applied to the study of epilepsy. For example, Mullinger et al. reveal the critical importance of the accuracy of physical models if one is to optimize lead positioning in functional MRI with simultaneous EEG. Confirming with computer modeling and phantom measurements that lead positioning can have a substantial effect on the amplitude of the MRI gradient artifact present on the EEG, they optimized the positions in a novel cap design. However, whilst this substantially reduced gradient artifact amplitude on the phantom, it made things worse when used on human subjects. Thus, improvements in model accuracy are required if one is to make accurate predictions for the human context.

Reduction of artifact, particularly cardioballistic and non-periodic motion artifact, remains a challenge for off-the-shelf MRI-compatible EEG systems. However, for over a decade, the Jackson group in Melbourne has dealt well with this issue using insulated carbon-fiber artifact detectors, physically but not electrically attached to the scalp (Masterton et al., 2007). In the present Research Topic, they provide detailed instructions for building such detectors and interfacing them with a commercially available MRI-compatible EEG system (Abbott et al.). This team also previously developed event-related ICA (eICA), to map fMRI activity associated with inter-ictal events observed on EEG (Masterton et al., 2013b). The method is capable of distinguishing separate sub-networks characterized by differences in spatio-temporal response (Masterton et al., 2013a). The eICA approach frees one from assumptions regarding the shape of the time-course of the neuronal and haemodynamic response associated with inter-ictal activity (which can vary according to spike type, can vary from conventional models and may include pre-spike activity, Masterton et al., 2010; issues explored further in the present topic by Faizo et al.; Jacobs et al.). However, the effectiveness of eICA can be affected by fMRI noise or artifact. In the present Research Topic we see that application of a fully automated de-noising algorithm (SOCK) is now recommended, as it can substantially improve the quality of eICA results (Bhaganagarapu et al.).

The ability to detect activity associated with inter-ictal events can also be improved with faster image acquisition. Magnetic Resonance Encephalography (MREG) is a particularly fast fMRI acquisition method (TR = 100 ms) that achieves its speed using an under-sampled k-space trajectory (Zahneisen et al., 2012; Assländer et al., 2013). This has now been applied in conjunction with simultaneous EEG, to reveal that the negative fMRI response in the default-mode network is larger in temporal compared to extra-temporal epileptic spikes (Jacobs et al.).

The default mode network and its relationship to epileptiform activity is also examined in several other papers in this Research Topic. In a pilot fMRI connectivity study of Genetic Generalized Epilepsy and Temporal Lobe Epilepsy patients, Lopes et al. observed that intrinsic connectivity in portions of the default mode network appears to increase several seconds prior to the onset of inter-ictal discharges. The authors suggest that the default mode network connectivity may facilitate IED generation. This is plausible, although causality is difficult to establish and it is possible that something else drives both the connectivity and EEG changes (Abbott).

Complicating matters further is the question of what connectivity means. There are many ways in which connectivity can be assessed. Jones and colleagues have discovered that some of these do not necessarily correlate well with each other. They examined connectivity between measurements made with intracranial electrodes, connectivity assessed using simultaneous BOLD fMRI and intracranial electrode stimulation, connectivity between low-frequency voxel measures of fMRI activity, and a diffusion MRI measure of connectivity—an integrated diffusivity measure along a connecting pathway (Jones et al.). They found only mild correlation between these four measures, implying they assess quite different features of brain networks. More research in this domain would therefore be valuable.

Whatever the measure of connectivity utilized, most evidence of alterations in connectivity in epilepsy has been obtained from comparison of a group of patients with a group of healthy controls. However, a new method called *Detection of Abnormal Networks in Individuals* (DANI) is now proposed by Dansereau et al. This method is designed to detect the organization of brain activity in stable networks, which the authors call modularity. The conventional definition of modularity refers to the degree to which networks can be segregated into distinct communities, usually estimated by maximizing within-group nodal links, and minimizing between group links (Girvan and Newman, 2002; Rubinov and Sporns, 2010). Dansereau take a novel approach to this concept, instead evaluating the stability of each resting state network across replications of a bootstrapped clustering method (Bellec et al., 2010). In the DANI approach, the degree to which an individual's functional connectivity modular pattern deviates from a population of controls is quantified. Whilst application of the method to epilepsy patients is preliminary, significant changes were reported likely related to the epileptogenic focus in 5 of the 6 selected focal epilepsy patients studied. In several patients, modularity changes in regions distant from the focus were also observed, adding further evidence that the pervasive network effects of focal epilepsy can extend well-beyond the seizure onset zone.

When it comes to application of EEG-fMRI to detect the seizure onset zone, there is typically a trade-off between specificity and sensitivity, with the added complication that activity or network changes may also occur in brain regions other than the ictal onset zone. The distant activity may be due to activity propagation from the onset zone, pervasive changes in functional networks creating a “permissive state,” or in some cases might be the brain's attempt to prevent seizures. Specificity and sensitivity of EEG-fMRI to detect the ictal onset zone is explored by Tousseyn et al.. They determined how rates of true and false positives and negatives varied with voxel height and cluster size thresholds, both for the full statistical parametric map, and for the single cluster that contained the voxel of maximum statistical significance. The latter conferred the advantage of reducing positives remote from the seizure onset zone. As a result, it appeared to be more robust to variations in statistical threshold than analysis of the entire map. One needs to be cautious however, given the small numbers of patients studied, and the fact that the “optimal” settings were determined using receiver operator characteristic curves of the same study data. It remains to be seen how well this might generalize to a different study.

Perhaps the greatest potential for future advancement in EEG-fMRI is in methods to make the most of the all the information captured by each modality. This is highlighted by the work of Deligianni et al. demonstrating with a novel analysis framework the potential to obtain more information on the human functional connectome by utilizing EEG and fMRI together (Abbott; Deligianni et al.).

We hope that you enjoy this collection of papers providing a broad snapshot of advances in brain mapping methods and application to better understand epilepsy.

## Author Contributions

DA conceived and designed the work and wrote the first draft of the manuscript. JA, PC, DV, and GJ all contributed to the conception of the work and revised the draft critically for intellectual content.

### Conflict of Interest Statement

The authors declare that the research was conducted in the absence of any commercial or financial relationships that could be construed as a potential conflict of interest.
